# Social and Demographic Predictors of Gender Inequality Among Heterosexual Couples Expecting a Child in Central Kenya

**DOI:** 10.1007/s40609-019-00138-3

**Published:** 2019-01-11

**Authors:** Caroline J. Vrana-Diaz, Jeffrey E. Korte, Mulugeta Gebregziabher, Lauren Richey, Anbesaw Selassie, Michael Sweat, Anthony Gichangi

**Affiliations:** 1 Department of Public Health Sciences, Medical University of South Carolina, 135 Cannon Street, Suite 303, Charleston, SC 29425, USA; 2 Section of Infectious Disease, Department of Medicine, Louisiana State University Health Sciences Center, 1542 Tulane Avenue, Suite 331, New Orleans, LA 70112, USA; 3 Department of Psychiatry and Behavioral Sciences, Medical University of South Carolina, 176 Croghan Spur Road, Suite 104, Charleston, SC 29407, USA; 4 Jhpiego, Ring Road, Arlington block, Nairobi, Kenya

**Keywords:** Genderequality, Antenatal care, Sociodemographics, Heterosexual couples, Pregnancy, HIV

## Abstract

Imbalance of power and equality in sexual relationships is linked to health in various ways, including (1) reduced ability to get information or take action, (2) increased violence between partners, and (3) influence on the reduced use of health services. While there has been research assessing multiple social and economic variables related to gender inequality, studies have used many different definitions of gender inequality, and there is a lack of this research within a pregnancy context. Here, we attempt to identify social and economic predictors of gender inequality (measured by decision-making power and acceptance of intimate partner violence) within heterosexual couples expecting a child in central Kenya. We ran a secondary data analysis using data from a three-arm individually randomized controlled HIV self-testing intervention trial conducted in 14 antenatal clinics in central and eastern Kenya among 1410 women and their male partners. The analysis included Cochran Mantel-Haenszel, logistic regression, proportional odds models, and generalized linear mixed model (GLMM) framework to account for site-level clustering. Overall, we show that there are significant social and economic variables associated with acceptance of intimate partner violence including higher age, being married, “other” religion, lower partner education, higher wealth status, and variables associated with decision-making power including lower partner education and lack of equality in earnings. This study contributes to the literature on the influence of social and economic factors on gender inequality, especially in Kenya which has a high burden of HIV/AIDS. Our results show some areas to improve these specific factors (including education and employment opportunities) or create interventions for targeted populations to potentially improve gender equality in heterosexual pregnant couples in Kenya.

## Introduction

In most societies, including African societies, males have more power over females (Organization for Social Science Research in Eastern and Southern Africa 2013). This imbalance is associated with violent and risky behaviors that can have a negative impact on many aspects of health (MacPherson et al. 2014). The balance of power in sexual relationships is linked to sexual and reproductive health in various ways, including directly through reduced ability to get information or take action, increased violence between partners, and through its influence on the reduced use of health services (Blanc 2001). Low educational, occupational, and economic opportunities and large age gaps in relationships have been associated with gender inequality, albeit with many different definitions of gender inequality. In South Africa and Botswana, there were a few social and economic predictors that were associated with lower female gender equality (measured by the woman’s decreased ability to suggest condom use to their partners), which included the partner being at least 10 years older than the woman, partners who abused the women, and women who are economically dependent on their partners (Langen 2005). Higher education was positively associated with more equitable gender norms among both men and women in a study of people living with HIV in South Africa (Fladseth et al. 2015). In a separate study of women in South Africa, researchers found that when both partners had higher education, they were more likely to discuss HIV (a marker of high gender equality), while there being an age difference of more than 5 years between the partners was associated with low discussion of HIV (Jewkes et al. 2003). Furthermore, when the woman had higher education, she was more likely to suggest condom use to her partner (Jewkes et al. 2003).

According to the United Nations Human Rights Commission (UNHRC) Report, “maternal mortality and morbidity is a consequence of gender inequality, discrimination, health inequity and a failure to guarantee women’s human rights” (United Nations Human Rights Council 2011). Furthermore, a meta-analysis from studies in Africa found an overall prevalence of IPV during pregnancy of 15.2% (ranging from 2 to 57%) (Shamu et al. 2011). Therefore, it is necessary to study the upstream sociodemographic predictors of gender equality among pregnant women, as a vulnerable population. Studies assessing gender equality and maternal health have shown that the presence of restrictive gender norms negatively affects the use of maternal health services in four sub-Saharan African countries (Adjiwanou and Legrand 2014), and that women with low decision-making autonomy were more likely to be exposed to maternal health risk (Banda et al. 2017). Another study found protective associations of gender equality (high household decision-making and low acceptance of IPV) on both maternal and child health outcomes (Singh et al. 2015). However, there have not been many studies assessing sociodemographic variables associated with gender inequality within a pregnancy context.

In this study, we therefore sought to investigate the associations between sociodemographic variables and gender inequality (measured by positive attitudes towards intimate partner violence and lower decision-making power) within the unique context of heterosexual couples expecting a child in central Kenya.

## Methods

### Design and Study Population

These data stem from a three-arm individually randomized HIV self-testing intervention trial conducted in 14 clinics in central and eastern Kenya, with study information collected at baseline and a 3-month follow-up visit (Gichangi et al. 2018).

Briefly, women were eligible to participate in the study if they were pregnant, at least 18 years old, and attending an antenatal clinic (ANC) for the first time for this pregnancy. Further inclusion criteria included reported contact with their male partner at least once per week, if their male partner was either HIV negative or their status unknown at the time of the woman’s recruitment, and that their male partner had not tested for HIV in the past 3 months. Women were excluded if they were concerned about the potential risk of violence from their male partner if they brought up the topic of HIV testing due to safety concerns, but very few women were excluded for this reason. After the women provided informed consent, they were randomized into one of three arms: Arm 1, the standard Kenyan Ministry of Health card inviting the male partner to come to the health clinic for a discussion on family health but nothing mentioning HIV; Arm 2, an improved invitation card describing the benefits of male HIV testing to prevent mother-to-child transmission of HIV; and Arm 3, the improved invitation card plus the delivery of two OraQuick HIV self-testing (HST) kits to the woman with instructions for testing the male partner at home. All three arms completed a baseline questionnaire. Three months after enrollment, the women were interviewed to ascertain whether or not their male partner tested for HIV, and the method of testing, as well as other variables. The male partners were also contacted at 3 months, and those consenting for an interview were administered a questionnaire on sociodemographics and HIV testing history.

### Measurements

Social variables included the age of both the man and woman (categorized from a continuous variable based on distributional balance), mother’s education level, religion, mother’s employment status, marital status, partner’s education level, and partner’s employment status. Economic variables included equality in earnings (the proportion of household expenses met by the woman’s earnings: none, less than a third, a third to a half, and more than half) and wealth index (a composite measure of a household’s cumulative living standard, separated into four wealth quartiles) (The DHS Program Wealth Index 2017). The wealth index consisted of the following variables: main source of drinking water, type of toilet facility, sharing of toilet, type of fuel used for cooking, presence of modern appliances (electricity, solar panels, generator, radio, television, refrigerator, telephone), ownership of transportation (bicycle, motorcycle, car), material of the house floor and roof, ownership of land or a house, ownership of productive assets (e.g., cattle or a sewing machine), and cash savings. This wealth index was constructed by the International Demographic and Health Surveys Program and has been used in research performed in Kenya (The DHS Program Wealth Index 2017). Rasch modeling was performed in the original trial to create the wealth index and then was separated into four quartiles (lowest, second lowest, second highest, and highest) (Gichangi et al. 2018).

The two primary outcome variables used in this study are measures of gender equality—namely attitudes towards IPV and decision-making power. Attitudes towards IPV was measured by the male’s report for the validated Violence Domain of the Gender Equitable Scale, a 5 question scale regarding hypothetical violence towards women, with available answers on a 2-point scale, where 1 = agree and 3 = disagree. Scores across all questions were summed and categorized into three levels: high acceptance of IPV (score of 5–11), medium acceptance of IPV (score of 13), and low acceptance of IPV (score of 15), where the higher the score, the lower acceptance of IPV (i.e., higher support for gender norms) (Compendium of Gender Scales Gender Equitable Men (GEM) Scale n.d.). Decision-making power was measured by the woman’s report on decision making for major household purchases, daily household needs, and visiting family or relatives, with available answers of (1) Myself, (2) My partner or others, or (3) Jointly. Each response to the three questions was dichotomized, with a value of 1 if the woman reports that a decision was made by either herself or jointly, and 0 if the decision was made by her male partner or someone else. We then created an index by summing the three dichotomized responses, with a value of 0 if the woman made no decisions (no decision-making power), 1 if she made one or two decisions by herself or jointly (low decision-making power), and 2 if she made all three decisions by herself or jointly (high decision-making power).

### Data Analysis

We summarized data using descriptive statistics where mean/ SD were reported for continuous variables and proportions were reported for categorical variables. To make comparisons between groups, we used Cochran Mantel-Haenszel. Modeling was performed with a generalized linear mixed models (GLMMs) framework, accounting for site-level clustering (Breslow and Clayton 1993). We checked the proportional odds assumption using the score test for proportional odds given in logistic regression (Agresti 2013). The first set of analyses was gender equality as measured by decision-making power from the woman’s report, with a categorical nominal outcome (due to violation of the proportional odds assumption). We used logistic regression and GLMM to estimate the odds ratio (OR) and corresponding 95% CI. The second was gender equality as measured by attitudes towards IPV from the man’s report (with an ordinal outcome), and we used cumulative logit and GLMM to estimate the parameters of the model. The intraclass correlation coefficient (ICC) was calculated to determine variation by clinic site. We chose our final model for each analysis based on a combination of factors including conceptual plausibility, individual variable significance in the model, confounding effect (including multicollinearity concerns), and two measures of model fit (Akaike’s Information Criterion and − 2 Log Likelihood, when appropriate). A two-sided p value of < 0.05 for specific variables was used to assess the significance of specific variables, as well as 95% CI not including 1. Proc GLIMMIX in SAS 9.4 (SAS Institute, Cary, NC) was used for all analyses.

### Ethical Approval

The original trial was approved by the institutional review board of the Kenya Research Medical Institute (IRB no. 485). Written informed consent was obtained from all participants. The current data analysis was performed on completely de-identified data and was deemed by the institutional review board of the Medical University of South Carolina as not human subjects research.

## Results

Table 1 shows the demographic characteristics of the women and their male partners. Overall, 1410 women were enrolled and randomized into the study, and 1217 women were interviewed at the 3-month follow-up visit. The original study attempted to reach all 1410 male partners, and 1130 male partners were interviewed at the 3-month follow-up visit. Male partners were on average older than the women (31.4 years versus 26.4 years, respectively), and in 84.7% of the relationships, the man was older than the woman. For women, the majority had a primary or lower education (56.1%), were mostly Protestant or other Christian besides Catholic (77.7%), were mostly self-employed (51.0%), were currently married (87.0%), had less than a third or none of the household expenses met by their earnings (65.5%), and the vast majority were HIV negative (96.2%). For the men, the majority had a secondary or higher education (60.1%), were mostly Protestant or other Christian (67.0%), were either employed for wages or self-employed (43.8% and 48.8%, respectively), were currently married (88.8%), and the vast majority were HIV negative (98.5%). The variables that were significantly different between male and female partners were age, education, religion, and employment. Overall, 22.2% of the men showed high support for hypothetical IPV, 22.2% had moderate support for IPV, and 55.6% had low support for IPV. For decision-making power, 12.8% of the women had no decision-making power, 32.3% of the women had low decision-making power, and 54.9% had high decision-making power.

**Table 1:** Characteristics of women attending antenatal care at baseline and characteristics of male partners at month 3 in Central Kenya

Characteristic		Women(n = 1410), n (%)		Male partners(n = 1130), n (%)
Age (years), mean ± SD*		26.4 ± 5.4		31.4 ± 5.6
Missing		0		18
Age categories*				
18-22 (women), 18-28 (men)		382 (27.1)		349 (31.4)
23-26 (women), 29-31 (men)		409 (29.1)		278 (25.0)
27-30 (women), 32-35 (men)		320 (22.7)		260 (23.4)
31-15 (women), 36-64 (men)		299 (21.2)		225 (20.2)
Age discrepancy between partners				
Same age or woman is older		170 (15.3)		–
Man is 1 -5 years older		448 (40.3)		–
Man is 6-10 years older		336 (30.2)		–
Man is 11+ years older		158 (14.2)		–
Missing		298		
Level of education*				
Primary or lower		791 (56.1)		449 (39.9)
Secondary or higher		619 (43.9)		677 (60.1)
Missing				4
Religion*				
Catholic		293 (20.78)		334 (29.6)
Protestant/other Christian		1096 (77.7)		757 (67.0)
Other		21 (1.5)		38 (3.4)
Missing				1
Employment status*				
Employed for wages		227 (16.1)		495 (43.8)
Self-employed		719 (51.0)		551 (48.8)
Not employed		464 (32.9)		83 (7.4)
Missing				1
Marital status				
Currently married		1227 (87.0)		1002 (88.8)
Not married		183 (12.98)		126 (11.2)
Missing		0		2
Proportion of expenses met by woman's earnings				
None		575 (40.8)		–
Less than a third		334 (23.7)		–
A third to a half		359 (25.5)		–
More than a half		141 (10.0)		–
Missing		1		
Wealth status				–
Lowest		300 (24.7)		–
Second lowest		306 (25.2)		–
Second highest		304 (25.0)		–
Highest		305 (25.1)		–
Missing		195		
HIV status				
Positive		9 (0.68)		6 (0.6)
Negative		1277 (96.2)		966 (98.5)
Indeterminate		14(1.1)		–
I did not receive result		28 (2.1)		–
Do not remember/do not wish to say		–		9 (0.9)
Missing		82		149
Intervention arm				
Standard of care		471 (33.4)		374 (33.1)
Improved invitation letter		467 (33.1)		361 (31.9)
HIV self-testing kits		472 (33.5)		395 (35.0)
Health facility				
Embu PGH		168 (11.9)		82 (7.3)
Githunguri Health Center		108 (7.7)		96 (8.5)
Kangeta Health Center		84 (6.0)		82 (7.3)
Kanyakini District Hospital		51 (3.6)		50 (4.4)
Kihara Sub-District Hospital		126 (8.9)		106 (9.4)
Kiritiri Health Center		63 (4.5)		58 (5.1)
Lari Health Center		91 (6.5)		71 (6.3)
Maragua District Hospital		96 (6.8)		89 (7.9)
Mbeere District Hospital		84 (6.0)		65 (5.8)
Meru Level 5 Hospital		207 (14.7)		188 (16.6)
Muthale Mission Hospital		70 (5.0)		62 (5.5)
Nyambene District Hospital		121 (8.6)		110 (9.7)
Tigoni District Hospital		75 (5.3)		25 (2.2)
Uthiru Health Center		66 (4.7)		46 (4.1)

SD, standard deviation

Columns may not total to 100 due to missing values

* p value for comparisons between female and male partners is < 0.05

Regarding the bivariate analyses between the demographic characteristics and gender equality, we found that lower scores on the Gender Equitable scale (i.e., high acceptance of IPV) were significantly associated with the following sociodemographics: the man being 11+ years older than the woman, primary or lower for women’s education, primary or lower for man’s education, man’s employment of out of work or self-employed, currently married, woman’s HIV status of positive or did not receive results, and higher wealth status. Lower decision-making power was significantly associated with lower women’s age, lower men’s age, lower men’s education, women out of work, man’s employment as self-employed, unmarried couples, low wealth status, and woman’s HIV status as did not receive results.

Table 2 shows the modeling of attitudes towards intimate partner violence by sociodemographics. The significant sociodemographic variables for this model were partner age, marital status, partner religion, partner education, wealth status, and woman’s HIV status. Specifically, compared to partners who were 18–28 years old, those who were 32–35 years old were more likely to indicate higher acceptance of IPV (OR, 1.81; 95% CI, 1.19–2.75). Unmarried persons had lower odds of increasing acceptance of IPV compared to married persons (OR, 0.59; 95% CI, 0.35–0.97). Partners who reported “other” religion were much more likely to have higher acceptance of IPV compared to those who were Protestant/other Christian (OR, 4.75; 95% CI, 2.14–10.53). Partners with a secondary or higher education had lower odds of acceptance of IPV compared to primary or lower education (OR, 0.42; 95% CI, 0.31–0.59). Those with the second highest or highest wealth status were more likely to have higher acceptance for IPV compared to the lowest wealth status (OR, 1.89; 95% CI, 1.20–2.99 and OR, 1.70; 95% CI, 1.02–2.82, respectively). Partners were more likely to have higher acceptance of IPV if the woman did not receive her last HIV test result compared to a negative test result (OR, 6.39; 95% CI, 1.90–21.42).

**Table 2:** Multivariate modeling for the ordinal outcome of gender inequality (measured by attitudes towards intimate partner violence)

	Attitudes towards intimate partner violence OR (95% CI)
Partner’s age (ref = 18–28)	
29–31	1.22 (0.81–1.84)
32–35	1.81 (1.19–2.75)*
36–64	1.39 (0.87–2.23)
Woman’s age (ref = 18–22)	
23–26	1.07 (0.73–1.57)
27–30	0.83 (0.53–1.29)
31–45	0.83 (0.51–1.34)
Marital status (ref = married)	
Not married	0.59 (0.35–0.97)*
Partner religion (ref = Protestant/other Christian)	
Catholic	1.06 (0.76–1.47)
Other	4.75 (2.14–10.53)*
Partner education (ref = primary or lower)	0.42 (0.31–0.59)*
Proportion of expenses met by woman’s earnings (ref = none)	
Less than one-third	0.84 (0.57–1.24)
One-third to one-half	0.95 (0.64–1.40)
More than one-half	1.39 (0.85–2.28)
Wealth status (ref = lowest)	
Second lowest	1.13 (0.75–1.72)
Second highest	1.89 (1.20–2.99)*
Highest	1.70 (1.02–2.82)*
Woman baseline HIV status (ref = negative)	
Positive	2.83 (0.31–25.54)
Indeterminate	0.42 (0.04–4.21)
Did not receive results	6.39 (1.90–21.42)*
Partner HIV status (ref = negative)	
Positive	0.21 (0.03–1.71)
Do not remember/did not want to say	2.28 (0.40–12.95)

* 95% CI does not include 1

Table 3 shows the modeling of decision-making power by sociodemographics. The significant sociodemographics include partner education and equality in earnings. Compared to a primary or lower education, partners who had a secondary or higher education were associated with lower odds of the woman having low decision-making power compared to high decision-making power (OR, 0.66; 0.46–0.93). Compared to women who met none of the household expenses with their earnings, women who met a third to a half of their household expenses by their earnings were less likely to have low decision-making power compared to high decision-making power (OR, 0.60; 0.37–0.95).

**Table 3:** Multivariate modeling for the nominal outcome of gender inequality (as measured by decision-making power, reference group = high)

	Decision-making power
Partner age (ref = 18–28) 29–31,	
outcome no power	1.31 (0.67–2.54)
29–31, outcome low power	1.13 (0.73–1.76)
32–35, outcome no power	0.99 (0.49–1.97)
35–35, outcome low power	0.82 (0.52–1.28)
36–64, outcome no power	0.47 (0.19–1.20)
36–64, outcome low power	0.62 (0.37–1.03)
Woman’s age (ref = 18–22)	
23–26, outcome no power	0.53 (0.28–1.02)
23–26, outcome low power	0.73 (0.48–1.12)
27–30, outcome no power	0.74 (0.36–1.54)
27–30, outcome low power	0.89 (0.55–1.43)
31–45, outcome no power	0.59 (0.25–1.36)
31–45, outcome low power	0.78 (0.46–1.34)
Marital status (ref = married)	
Not married, outcome no power	1.67 (0.71–3.91)
Not married, outcome low power	1.33 (0.79–2.28)
Partner education (ref = primary or lower)	
Secondary or higher, outcome no power	0.77 (0.45–1.34)
Secondary or higher, outcome low power	0.66 (0.46–0.93)*
Proportion of expenses met by woman’s earnings (ref = none)	
Less than one-third, outcome no power	0.58 (0.27–1.23)
Less than one-third, outcome low power	0.89 (0.57–1.36)
One-third to one-half, outcome no power	0.56 (0.27–1.17)
One-third to one-half, outcome low power	0.60 (0.37–0.95)*
More than one-half, outcome no power	0.39 (0.14–1.05)
More than one-half, outcome low power	0.53 (0.28–1.01)
Wealth status (ref = lowest)	
Second lowest, outcome no power	0.85 (0.44–1.66)
Second lowest, outcome low power	0.74 (0.46–1.20)
Second highest, outcome no power	0.55 (0.24–1.27)
Second highest, outcome low power	0.77 (0.47–1.25)
Highest, outcome no power	0.51 (0.20–1.34)
Highest, outcome low power	0.61 (0.35–1.06)
Woman employment (ref = employed for wages)	
Out of work (unemployed), outcome no power	1.00 (0.47–2.13)
Out of work (unemployed), outcome low power	1.02 (0.61–1.70)
Self-employed, outcome no power	0.56 (0.25–1.26)
Self-employed, outcome low power	1.30 (0.79–2.14)

* 95% CI does not include 1

## Discussion

This study was conducted in order to identify social and economic predictors of gender inequality (measured by gender power imbalance and positive attitudes towards IPV) among heterosexual couples expecting a child in central Kenya within the context of a HIV self-testing randomized controlled trial. Overall, we found higher acceptance of intimate partner violence among (a) partners with lower education, (b) married, (c) religion other than Christian, (d) partner’s with higher age, (e) higher wealth status, and (f) woman not receiving HIV test results. In addition, we found lower decision-making power among (a) partners with lower education, and (b) those with a lack of equality in earnings.

We found that partners with secondary education or higher were less likely to have higher acceptance of intimate partner violence compared to those with primary education or lower, as well as less likely to have a woman with low decision-making power. This is consistent with studies showing that secondary or higher education is consistently associated with high support for gender equality in men (Levtov et al. 2014; Slegh et al. 2014; Lusey et al. 2017) and associated with reduced IPV (Vakili et al. 2010; Osinde et al. 2011; Capaldi et al. 2012). Those who self-identified as “other” religion were much more likely to have higher acceptance of IPV compared to Protestant/other Christian. A study in Bangladesh that found women who were Muslim were more likely to think that IPV was justified compared to any other religion (Biswas et al. 2017), and one in Ghana showing that women who were Muslim and Traditional believers were more likely to approve domestic physical violence compared to women who were Christian (Doku and Asante 2015). Partners who were 32–35 were more likely to have higher acceptance of IPV compared to male partners who were younger (18–28 years old). This is opposite from a systematic review showing a negative association of age and IPV, although this systematic review detailed perpetration of IPV, not acceptance of IPVas was described in this current analysis (Capaldi et al. 2012), but is in line with a study in South Africa reporting that higher age was negatively associated with more equitable gender norms in both men and women (Fladseth et al. 2015). Those with higher wealth status (second highest or highest quartile) were more likely to have higher acceptance of IPV compared to the lowest quintile. This is opposite from many studies that show that high income is associated with less IPV or less justification of IPV (Rani et al. n.d.; Vakili et al. 2010; Doku and Asante 2015; Biswas et al. 2017). In our study, those that were unmarried were less likely to report higher support for intimate partner violence compared to those who were married. Research in the Democratic Republic of the Congo found that men who were unmarried or separated had higher support for gender equality than those who were married (Lusey et al. 2017). Women who contributed a third to a half of household expenses with their own earnings (compared to none) were less likely to have low decision-making power compared to high decision-making power. This is consistent with a study that showed women who were economically dependent on their partners had lower gender equality (Langen 2005).

**Limitations** There are a couple of noteworthy limitations in this study. First, women were excluded if they were concerned about violence from their male partner if they were to bring home the HIV self-testing kit. This was expected to bias our study population to include those with less intimate partner violence than the general population. However, the participation rates from eligible women in the original trial were very high, so very few women self-excluded from participating due to fear of IPV. Furthermore, there could have been residual confounders that impacted our observed associations due to uncollected measurements.

## Conclusions

In summary, higher partner’s age, lower woman’s age, marriage, “other” or Catholic religion, lower partner’s education, higher wealth status, and lack of equality in earnings were found to be associated with gender inequality. This study contributes to the literature on the influence of social and economic factors on gender inequality, especially in the country of Kenya and in an HIV-related and pregnancy context. These results show some promising areas to target to improve these specific social and economic variables (especially to increase partner education levels and increase equality in earnings between partners) or create interventions to targeted populations (specifically targeted towards different religions or wealth statuses, or married couples) to potentially improve gender equality in heterosexual couples expecting a child in Kenya.

## References

[cit0001] Adjiwanou V., & Legrand T (2014). Gender inequality and the use of maternal healthcare services in rural sub-Saharan Africa. *Health & Place*. 10.1016/j.healthplace.2014.06.001 24994096

[cit0002] Agresti A (2013). In 3rd (Ed.), *Categorical data analysis*. Hobboken: John Wiley Sons.

[cit0003] Banda P. C., Odimegwu C. O., Ntoimo L. F. C., & Muchiri E (2017). Women at risk: gender inequality and maternal health. *Women & Health*, 57(4), 405–429. 10.1080/03630242.2016.1170092. 27015080

[cit0004] Biswas R. K., Rahman N., Kabir E., & Raihan F (2017). Women’s opinion on the justification of physical spousal violence: a quantitative approach to model the most vulnerable households in Bangladesh. *PLoS One*, 12(11), e0187884 10.1371/journal.pone.0187884. 29161277 PMC5697832

[cit0005] Blanc A. K (2001). The effect of power in sexual relationships on sexual and reproductive health: an examination of the evidence. *Studies in Family Planning*, 32(3), 189–213. 11677692 10.1111/j.1728-4465.2001.00189.x

[cit0006] Breslow N. E., & Clayton D. G (1993). Approximate inference in generalized linear mixed models. *Journal of the American Statistical Association*, 88(421), 9 10.2307/2290687.

[cit0007] Capaldi D. M., Knoble N. B., Shortt J. W., & Kim H. K (2012). A systematic review of risk factors for intimate partner violence. *Partner Abuse*, 3(2), 231–280. 10.1891/1946-6560.3.2.231. 22754606 PMC3384540

[cit0008] Compendium of Gender Scales Gender Equitable Men (GEM) Scale (n.d.)..

[cit0009] Doku D. T., & Asante K. O (2015). Women’s approval of domestic physical violence against wives: analysis of the Ghana demographic and health survey. *BMC Womens Health*, 15(1), 120 10.1186/s12905-015-0276-0. 26691763 PMC4687112

[cit0010] Fladseth K., Gafos M., Newell M. L., & Mcgrath N (2015). The impact of gender norms on condom use among HIV-positive adults in KwaZulu-Natal, South Africa. *PLoS One*, 10(4), 122671 10.1371/journal.pone.0122671. PMC4390283 25853870

[cit0011] Gichangi A., Wambua J., Mutwiwa S., Njogu R., Bazant E., Wamicwe J., Wafula R., Vrana C. J., Stevens D. R., Mudany M., & Korte J. E (2018). Impact of HIV self-test distribution to male partners of ANC clients. *Journal of Acquired Immune Deficiency Syndromes*, 74, 467–473. 10.1097/QAI.0000000000001838. PMC6250253 30148731

[cit0012] Jewkes R. K., Levin J. B., & Penn-Kekana L. A (2003). Gender inequalities, intimate partner violence and HIV preventive practices: findings of a south African cross-sectional study. *Social Science & Medicine*, 56(1), 125–134. 12435556 10.1016/s0277-9536(02)00012-6

[cit0013] Langen T. T (2005). Gender power imbalance on women’s capacity to negotiate self-protection against HIV/AIDS in Botswana and South Africa. *African Health Sciences*, 5(3), 188–197. 10.5555/afhs.2005.5.3.188. 16245988 PMC1831928

[cit0014] Levtov R. G., Barker G., Contreras-Urbina M., Heilman B., & Verma R (2014). Pathways to gender-equitable men: findings from the International Men and Gender Equality Survey in eight countries. *Men and Masculinities*, 17, 467–501. 10.1177/1097184X14558234.

[cit0015] Lusey H., San Sebastian M., Christianson M., & Edin K. E (2017). Factors associated with gender equality among church-going young men in Kinshasa, Democratic Republic of Congo: a cross-sectional study. *International Journal for Equity in Health*, 16(1), 213 10.1186/s12939-017-0707-7. 29228996 PMC5725947

[cit0016] MacPherson E. E., Richards E., Namakhoma I., & Theobald S (2014). Gender equity and sexual and reproductive health in eastern and southern Africa: a critical overview of the literature. *Global Health Action*, 7(1), 1–9. 10.3402/gha.v7.23717. PMC4074359 24972916

[cit0017] Organization for Social Science Research in Eastern and Southern Africa (2013). *Insights into gender equity, equality, and power relations in sub-Saharan Africa*. Kampala: Fountain Publishers.

[cit0018] Osinde M. O., Kaye D. K., & Kakaire O (2011). Intimate partner violence among women with HIVinfection in rural Uganda: critical implications for policy and practice. *BMC Womens Health*, 11(50). 10.1186/1472-6874-11-50. PMC3231867 22093904

[cit0019] Rani M., Bonu S., Diop-Sidibé N (2004). An empirical investigation of attitudes towards wife-beating among men and women in seven sub-Saharan African countries. *African Journal of Reproductive Health*, 8(3), 116–136. 17348330

[cit0020] Shamu S., Abrahams N., Temmerman M., Musekiwa A., & Zarowsky C (2011). A systematic review of African studies on intimate partner violence against pregnant women: prevalence and risk factors. *PLoS One*, 6(3), e17591 10.1371/journal.pone.0017591. 21408120 PMC3050907

[cit0021] Singh K., Bloom S., & Brodish P (2015). Gender equality as a means to improve maternal and child health in Africa. *HealthCare for Women International*, 36(1), 57–69. 10.1080/07399332.2013.824971. PMC4333143 24028632

[cit0022] Slegh H., Barker G., & Levtov R (2014). Gender relations, sexual and gender-based violence and the effects of conflict on women and men in North Kivu, Eastern Democratic Republic of the Congo: results from the International Men and Gender Equality Survey (IMAGES). Washington, DC, and Capetown, South Africa: Promundo-US and Sonke Gender Justice.

[cit0023] The DHS Program Wealth Index. http://www.dhsprogram.com/topics/wealth-index/Index.cfm. Accessed 19 Apr 2017.

[cit0024] United Nations Human Rights Council (2011) Practices in adopting a human rights-based approach to eliminate preventable maternal mortality and human rights.

[cit0025] Vakili M., Nadrian H., Fathipoor M., Boniadi F., Mohammad B. S., & Morowatisharifabad A (2010). Prevalence and determinants of intimate partner violence against women in Kazeroon, Islamic Republic of Iran. *Violence and Victims*, 25(1), 116–127. 10.1891/0886-6708.25.1.116. 20229697

