# Supplementary material for: Social and Demographic Predictors of Gender Inequality Among Heterosexual Couples Expecting a Child in Central Kenya
Source: Glob Soc Welf. 2019 Jan 11:10.1007/s40609-019-00138-3. Online ahead of print. doi: 10.1007/s40609-019-00138-3 (PMC7734197; doi:10.1007/s40609-019-00138-3)
Supplement: Supplementary file 1 [file GSW-2019-001383-s001.docx]

**Supplemental Table 1.** Bivariate analysis of sociodemographic characteristics of women and male partners and gender equality

|  | **Attitudes towards IPV** | | | **Decision-Making Power** | | |
| --- | --- | --- | --- | --- | --- | --- |
|  | **High support for IPV** | **Medium support for IPV** | **Low support for IPV** | **None** | **Low** | **High** |
| **Sociodemographics, n (%)** |  |  |  |  |  |  |
| Women age categories**^^ |  |  |  |  |  |  |
| 18-22 | 72 (22.9) | 78 (24.7) | 165 (52.4) | 69 (18.1) | 127 (33.3) | 186 (48.7) |
| 23-26 | 73 (22.4) | 66 (20.3) | 187 (57.4) | 52 (12.7) | 133 (32.5) | 224 (54.8) |
| 27-30 | 50 (19.7) | 55 (21.6) | 149 (58.7) | 36 (11.3) | 106 (33.1) | 178 (55.6) |
| 31-45 | 55 (23.8) | 51 (22.1) | 125 (54.1) | 23 (7.7) | 90 (30.1) | 186 (62.2) |
| Men age categories**^^ |  |  |  |  |  |  |
| 18-28 | 87 (25.1) | 60 (17.3) | 200 (57.6) | 37 (10.6) | 122 (35.0) | 190 (54.4) |
| 29-31 | 39 (14.1) | 77 (27.8) | 161 (58.1) | 57 (20.5) | 80 (28.8) | 141 (50.7) |
| 32-35 | 59 (22.7) | 58 (22.3) | 143 (55.0) | 34 (13.1) | 71 (27.3) | 155 (59.6) |
| 36-64 | 62 (27.6) | 54 (24.0) | 109 (48.4) | 10 (4.4) | 62 (27.6) | 153 (68.0) |
| Age Discrepancy between partners |  |  |  |  |  |  |
| Same age or woman is older | 29 (17.3) | 36 (21.6) | 102 (61.0) | 25 (14.7) | 47 (27.6) | 98 (57.6) |
| Man is 1-5 years older | 98 (21.9) | 87 (19.4) | 263 (58.7) | 46 (10.3) | 148 (33.0) | 254 (56.7) |
| Man is 6-10 years older | 76 (22.6) | 83 (24.7) | 177 (52.7) | 44 (13.1) | 104 (30.9) | 188 (55.9) |
| Man is 11+ years older | 44 (27.8) | 43 (27.2) | 71 (44.9) | 23 (14.6) | 36 (22.8) | 99 (62.7) |
| Women Level of education |  |  |  |  |  |  |
| Primary | 167 (25.4) | 148 (22.5) | 342 (52.0) | 101 (12.8) | 257 (32.5) | 433 (54.7) |
| Secondary (A or O level) | 83 (17.7) | 102 (21.8) | 284 (60.5) | 79 (12.8) | 199 (32.2) | 341 (55.1) |
| Man Level of Education**^ |  |  |  |  |  |  |
| Primary or Lower | 148 (33.0) | 120 (26.7) | 181 (40.3) | 65 (14.5) | 146 (32.5) | 238 (53.0) |
| Secondary or Higher | 101 (15.0) | 129 (19.1) | 444 (65.9) | 73 (10.8) | 196 (28.9) | 408 (60.3) |
| Religion |  |  |  |  |  |  |
| Catholic | 55 (23.6) | 47 (20.2) | 131 (56.2) | 40 (13.6) | 90 (30.7) | 163 (55.6) |
| Protestant/other Christian | 190 (21.7) | 198 (22.6) | 489 (55.8) | 138 (12.6) | 361 (32.9) | 597 (54.5) |
| Other | 5 (31.3) | 5 (31.3) | 6 (37.5) | 2 (9.5) | 5 (23.8) | 14 (66.7) |
| Women Employment^^ |  |  |  |  |  |  |
| Employed for wages | 40 (22.5) | 31 (17.4) | 107 (60.1) | 33 (14.5) | 73 (32.2) | 121 (53.3) |
| Self-employed | 135 (22.7) | 128 (21.5) | 187 (53.0) | 39 (8.4) | 152 (32.8) | 273 (58.8) |
| Out of work | 75 (21.3) | 91 (25.8) | 332 (55.8) | 108 (15.0) | 231 (32.1) | 380 (52.8) |
| Men Employment^ |  |  |  |  |  |  |
| Employed for wages | 94 (19.0) | 106 (21.5) | 294 (59.5) | 50 (10.1) | 153 (30.9) | 292 (59.0) |
| Self-employed | 132 (24.0) | 131 (23.9) | 286 (52.1) | 84 (15.3) | 163 (29.6) | 304 (55.2) |
| Student/Out of Work | 24 (31.2) | 13 (15.7) | 46 (55.4) | 5 (6.0) | 26 (31.3) | 52 (62.6) |
| Marital status**^^ |  |  |  |  |  |  |
| Currently married | 231 (24.0) | 223 (23.1) | 509 (52.9) | 155 (12.6) | 377 (30.7) | 695 (56.6) |
| Not Married | 19 (11.7) | 27 (16.6) | 117 (71.8) | 25 (13.7) | 79 (43.2) | 79 (43.2) |
| Woman HIV status**^ |  |  |  |  |  |  |
| Positive | 4 (66.7) | 0 (0) | 2 (33.3) | 0 (0) | 4 (44.4) | 5 (55.6) |
| Negative | 227 (22.1) | 226 (22.0) | 576 (56.0) | 157 (12.3) | 417 (32.6) | 703 (55.0) |
| Indeterminate | 1 (9.1) | 1 (9.1) | 8 (81.8) | 0 (0) | 3 (21.4) | 11 (78.6) |
| Did not receive result | 11 (55.0) | 4 (20.0) | 5 (25.0) | 7 (25.0) | 12 (42.9) | 9 (32.1) |
| Man HIV status |  |  |  |  |  |  |
| Positive | 1 (16.7) | 1 (16.7) | 4 (66.7) | 0 (0) | 2 (33.3) | 4 (66.7) |
| Negative | 209 (21.6) | 216 (22.4) | 540 (56.0) | 133 (13.8) | 280 (29.0) | 553 (57.3) |
| Don’t wish to say | 4 (44.4) | 1 (11.1) | 4 (44.4) | 1 (11.1) | 2 (22.2) | 6 (66.7) |
| Wealth Status*^^ |  |  |  |  |  |  |
| Lowest | 43 (15.7) | 57 (20.8) | 173 (63.4) | 44 (14.7) | 105 (35.0) | 151 (50.3) |
| Second Lowest | 43 (15.7) | 77 (28.1) | 154 (56.2) | 72 (23.5) | 85 (27.8) | 149 (48.7) |
| Second Highest | 70 (25.0) | 61 (21.8) | 149 (53.2) | 19 (6.3) | 107 (35.2) | 178 (58.6) |
| Highest | 90 (32.3) | 51 (18.3) | 138 (49.5) | 15 (4.9) | 86 (28.2) | 204 (66.9) |
| Proportion of expenses met by woman’s earnings**^ |  |  |  |  |  |  |
| None | 112 (25.1) | 94 (21.0) | 241 (53.9) | 79 (13.7) | 212 (36.9) | 284 (49.4) |
| Less than a third | 50 (18.2) | 61 (22.2) | 164 (59.6) | 27 (8.1) | 113 (33.8) | 194 (58.1) |
| A third to a half | 59 (20.6) | 61 (21.3) | 167 (58.2) | 44 (12.3) | 96 (26.7) | 219 (61.0) |
| More than a half | 28 (24.1) | 34 (29.3) | 54 (46.6) | 30 (21.3) | 34 (24.1) | 77 (54.6) |
| Attitudes towards IPV: *p<0.05, **p< 0.01, Decision-Making Power: ^p<0.05, ^^p<.01 | | | | | | |
